# Type 2 diabetes mellitus in people with severe mental illness: inequalities by ethnicity and age. Cross‐sectional analysis of 588 408 records from the UK

**DOI:** 10.1111/dme.13298

**Published:** 2017-01-30

**Authors:** J. Das‐Munshi, M. Ashworth, M. E. Dewey, F. Gaughran, S. Hull, C. Morgan, J. Nazroo, I. Petersen, P. Schofield, R. Stewart, G. Thornicroft, M. J. Prince

**Affiliations:** ^1^ Department of Health Service and Population ResearchInstitute of PsychiatryPsychology & NeuroscienceKing's College LondonLondon; ^2^ Department of Primary Care and Public Health SciencesKing's College LondonLondon; ^3^ South London and Maudsley NHS Foundation TrustLondon; ^4^ Blizard InstituteBarts and London School of Medicine and DentistryLondon; ^5^ Cathie Marsh Institute for Social ResearchUniversity of ManchesterManchester; ^6^ Department of Primary Care and Population HealthUniversity College LondonLondonUK

## Abstract

**Aims:**

To investigate whether the association of severe mental illness with Type 2 diabetes varies by ethnicity and age.

**Methods:**

We conducted a cross‐sectional analysis of data from an ethnically diverse sample of 588 408 individuals aged ≥18 years, registered to 98% of general practices (primary care) in London, UK. The outcome of interest was prevalent Type 2 diabetes.

**Results:**

Relative to people without severe mental illness, the relative risk of Type 2 diabetes in people with severe mental illness was greatest in the youngest age groups. In the white British group the relative risks were 9.99 (95% CI 5.34, 18.69) in those aged 18–34 years, 2.89 (95% CI 2.43, 3.45) in those aged 35–54 years and 1.16 (95% CI 1.04, 1.30) in those aged ≥55 years, with similar trends across all ethnic minority groups. Additional adjustment for anti‐psychotic prescriptions only marginally attenuated the associations. Assessment of estimated prevalence of Type 2 diabetes in severe mental illness by ethnicity (absolute measures of effect) indicated that the association between severe mental illness and Type 2 diabetes was more marked in ethnic minorities than in the white British group with severe mental illness, especially for Indian, Pakistani and Bangladeshi individuals with severe mental illness.

**Conclusions:**

The relative risk of Type 2 diabetes is elevated in younger populations. Most associations persisted despite adjustment for anti‐psychotic prescriptions. Ethnic minority groups had a higher prevalence of Type 2 diabetes in the presence of severe mental illness. Future research and policy, particularly with respect to screening and clinical care for Type 2 diabetes in populations with severe mental illness, should take these findings into account.

## What's new?

- There is limited evidence of the association of severe mental illness with Type 2 diabetes mellitus in ethnic minorities.
- Using data from >500 000 people, we established that: (1) risk of Type 2 diabetes was increased up to 10‐fold in people with severe mental illness compared with groups without severe mental illness, irrespective of ethnicity, and was greatest in the youngest age groups; (2) prevalence of Type 2 diabetes was highest in Bangladeshi people with severe mental illness but was also high in all other South Asian, black African and black Caribbean groups; and (3) most associations persisted despite adjustment for anti‐psychotic prescriptions.

**What's new?**

- There is limited evidence of the association of severe mental illness with Type 2 diabetes mellitus in ethnic minorities.
- Using data from >500 000 people, we established that: (1) risk of Type 2 diabetes was increased up to 10‐fold in people with severe mental illness compared with groups without severe mental illness, irrespective of ethnicity, and was greatest in the youngest age groups; (2) prevalence of Type 2 diabetes was highest in Bangladeshi people with severe mental illness but was also high in all other South Asian, black African and black Caribbean groups; and (3) most associations persisted despite adjustment for anti‐psychotic prescriptions.

## Introduction

Life expectancy in people with severe mental illness, such as schizophrenia, bipolar affective disorder and other non‐organic psychoses, is reduced by 15–20 years compared with the general population 1. A large proportion of these deaths are accounted for by natural causes 2. At least one third of the reduction in life expectancy is attributable to cardiovascular mortality 3. Associated with this, the prevalence of Type 2 diabetes mellitus is estimated to be two‐ to threefold higher in people with severe mental illness compared with the general population 4, with overall prevalence estimated to be between 1.26 and 50% 5. Proposed mechanisms include impact of medications such as anti‐psychotic drugs 5, 6, 7, social deprivation and lifestyle 5, 8, as well as the direct effect of severe mental illness through chronic stress 8 or mediated through changes in inflammatory markers and the hypothalamic–pituitary–adrenal axis 4.

Some ethnic minority groups, such as black or Hispanic people may be at a higher risk of Type 2 diabetes mellitus if also diagnosed with severe mental illness 5, 9. Much of the research in this area has been based on non‐epidemiological convenience samples from psychiatric clinics 9. Irrespective of the presence of severe mental illness, high prevalence of Type 2 diabetes mellitus have been reported in other ethnic minority groups, including Bangladeshi, Pakistani, Indian, black Caribbean and black African populations 10, 11, 12. No study has systematically assessed the prevalence of Type 2 diabetes mellitus in these groups when also diagnosed with severe mental illness.

With this in mind, the aim of the present study was to assess the association of severe mental illness with diabetes mellitus, using a large cross‐sectional dataset of patient records from UK primary care. Practices were located in an ethnically diverse urban location, where many ethnic minority people reside and where the incidence of severe mental illness is elevated 13. We hypothesized that the prevalence of Type 2 diabetes mellitus in people with severe mental illness would be more elevated in ethnic minority groups already known to be at an increased risk of Type 2 diabetes mellitus, specifically Indian, Pakistani, Bangladeshi, black Caribbean and black African people, compared with white British people 10, and that the added risk of living with diabetes mellitus and severe mental illness for these groups would be greater than for white British people with severe mental illness and would persist after taking into account anti‐psychotic prescriptions, which are known to increase the risk of Type 2 diabetes mellitus in populations with severe mental illness. The present analysis is part of a larger study designed to investigate cardiovascular health inequalities in people with severe mental illness 13.

## Methods

### Design, setting and population

Data from individuals aged ≥18 years, registered to 189 of the 192 general practices (98%) in the London boroughs of Tower Hamlets, Newham, City of London, Hackney and Lambeth were used for the analyses. Each of these boroughs are resident to some of the largest ethnic minority communities in the UK, including Bangladeshi, black Caribbean and Black African communities; 51% of the population in the study area self‐identify as belonging to an ethnic minority group 14. All patient records for 1 year before the date of extraction were included in the analyses. This was 31 March 2013 for records from East London (Tower Hamlets, Newham, City of London and Hackney) and 31 October 2013 for records from Lambeth. Analyses were cross‐sectional; individuals were considered to have a severe mental illness, Type 2 diabetes mellitus or to be on an anti‐psychotic prescription if there was a record of this at any point in the observation period.

### Measures

In the UK, 95% of the population is registered with general practice. General practice is the first point of contact for the National Health Service (NHS) and allows patient access to family physicians, nurses or other community health staff 15. A pay‐for‐performance scheme, the Quality and Outcomes Framework (QOF), was established as part of the GP contract in 2004 16 and covers the care of all individuals registered to primary care in England 16. The QOF provides general practitioners (GPs) with a financial incentive to keep an up‐to‐date register of people with a confirmed diagnosis of schizophrenia, bipolar affective disorder and non‐organic psychosis 16 and means that people with these disorders are recognized and recorded more frequently in UK primary care 17. At the time of this study, GPs were incentivized to ensure that health checks in people with severe mental illness, including the assessment of HbA_1c_ and glucose measurement, were undertaken annually 16. Diagnostic Read codes 18 were used to derive main exposure and outcome measures used in the analysis. Read codes are a thesaurus of standardized clinical terms which provide the means through which clinicians record patient health indicators 18.

### Exposure

#### Severe mental illness

Individuals with a diagnosis of schizophrenia, bipolar affective disorder or non‐organic psychosis were identified using diagnostic codes and grouped together to form the main exposure category of ‘severe mental illness’. The use of computer‐based electronic records to identify individuals with severe mental illness in UK primary care has previously been validated, with a sensitivity of 91% and a positive predictive value of 91% for non‐organic psychosis assessed against a syndrome checklist derived from the Present State Examination and International Classification of Disease‐9 (ICD‐9) codes 19. Recent work has shown that in UK primary care, this diagnostic grouping remains stable over time, and the incidence of severe mental illness in primary care is broadly similar to established epidemiological trends, with respect to gender, age and socio‐economic deprivation 17. Up to one third of people with severe mental illness may be registered with a GP but not known to secondary care 20.

### Outcome

**Figure 1:**
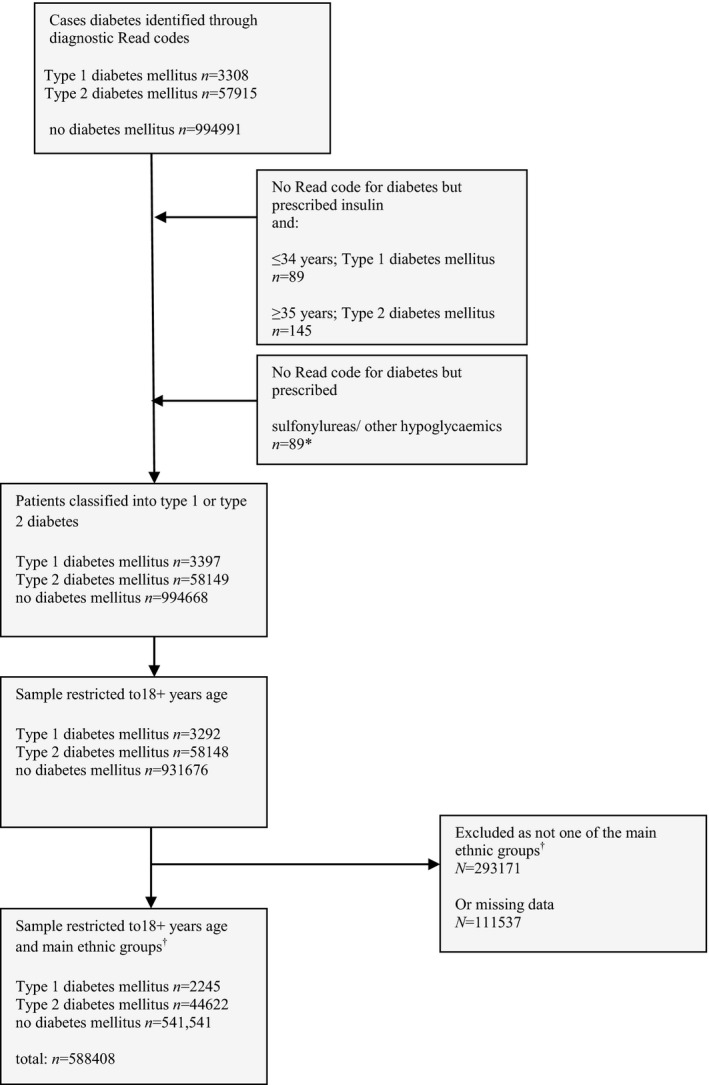
Flow chart of participants into study. *n = 764 people were prescribed Metformin only with no Read code for diabetes mellitus; (Metformin is prescribed for other conditions) these patients were not included in the Type 2 diabetes mellitus group; ^†^Main ethnic groups in the study: white British, Irish, Indian, Pakistani, Bangladeshi, black Caribbean and black African. Excluded ethnic minority groups were mainly ‘other’ ethnic groups.

Diagnoses of diabetes mellitus were ascertained by reviewing diagnostic codes 18 entered by GPs as well as reviewing entries on pharmacy records. A clinician (J.D.) manually reviewed all diagnostic codes. Criteria for diagnosis of diabetes mellitus were informed by approaches used in other primary care database studies of diabetes mellitus, such as the Health Improvement Network (THIN) 21 and the Clinical Practice Research Datalink (CPRD) 22. Figure 1 shows how Type 2 diabetes mellitus was determined.

### Effect modifiers and confounders

Age at last birthday and gender were available for all participants. Age was analysed as a continuous variable and then categorized into three groups (18–34, 35–54 and ≥55 years). This afforded sufficient numbers within each group to compare associations with Type 2 diabetes mellitus by ethnicity. Measures for area‐level deprivation were derived by mapping postcodes of participants to Lower Level Super Output Areas, which were then linked to the Index of Multiple Deprivation (IMD 2010) 23. Anti‐psychotic medications were assessed using data on prescriptions, leading to a binary variable (prescribed anti‐psychotics or not prescribed anti‐psychotics).

### Ethnicity

Across the study sites, the recording of self‐ascribed ethnicity has been promoted through locally run incentive schemes, with high levels of completeness on this variable. Self‐ascribed ethnicity mapped to the 2011 UK census categories was used and categorized using approaches similar to previous national surveys from England 10, 11. The resultant ethnic groups were: white British; Irish; Indian; Pakistani; Bangladeshi; black Caribbean; and black African. The Irish ethnicity group was retained as distinct to the white British group, as previous research has indicated poorer health outcomes in this group 24.

### Statistical analysis

Generalized linear models with log link and Poisson distribution 25 were used to derive relative risks 25. Equal follow‐up times were attributed to individuals in these models. Relative risks were chosen over odds ratios as the outcome (Type 2 diabetes mellitus) was relatively prevalent and, in these circumstances, odds ratios may overestimate the prevalence ratio 26. As the variation in binary data may be overestimated using Poisson regression, a robust variance estimator was initially used 25. The 95% CIs derived using this approach against approaches which used Poisson regression with robust standard errors to account for clustering by general practice were similar to three decimal places. Models were stratified by age and ethnicity and adjusted for (1) gender and area‐level deprivation and (2) gender, area‐level deprivation and anti‐psychotic prescriptions, with clustering by general practice accounted for through robust standard errors. This approach was used to assess the crude and adjusted association of severe mental illness with Type 2 diabetes mellitus, stratified by age and ethnicity, leading to relative risks with 95% CIs.

In keeping with reporting guidelines, and in order to provide a fuller assessment of potential inequalities, we also opted to assess absolute measures of effect 27. This approach complemented the relative risk‐based approach and allowed us to clarify differences in baseline risk of Type 2 diabetes mellitus in ethnic groups and the effect of also being diagnosed with severe mental illness (therefore leading to estimates with more direct relevance to clinical practice). Generalized linear models with a binomial distribution and an identity link were used to derive adjusted risk differences for Type 2 diabetes mellitus in people with severe mental illness relative to those without severe mental illness 28, stratified by ethnicity and age. The ‘margins’ command in stata was used to derive estimated prevalence from these models. Risk difference models were implemented in stata based on Wacholder's method 29. All models adjusted for gender, area‐level deprivation and robust standard errors to account for practice‐level clustering. Analyses were complete case. All statistical tests were two‐tailed. Analyses were conducted in stata 13 28.

### Ethical approval

The study was approved by Kings College London Research Ethics Committee. Locally, the South London Primary Care Research Governance Team reviewed the process of anonymized data analysis confirming that research governance assurance was not required. As a secondary analysis of anonymized data this study did not require national ethics approval. The dataset was constructed by pooling primary care data across boroughs; no data linkages were sought. The pooled dataset has contributed to several observational studies using anonymized data.

## Results

Data for age, gender, practice location and anti‐psychotic prescriptions were complete. There were 33 656 (6%) individuals without information on area‐level deprivation and 111 537 (11%) without information on ethnicity. After restricting the analysis to participants who could be mapped on to the main ethnic groups, data from 588 408 individuals, aged ≥18 years and registered to 189 general practices, were included in the analysis (Fig. 1). Table 1 shows the demographic features. Notably, slightly more people were prescribed anti‐psychotic medications than had a severe mental illness diagnosis (Table 1).

**Table 1:** Demographic features of the sample

Total sample, *N* (%)	588 408 (100)
Age group, *n* (%)	
18–34 years	250 883 (43)
35–54 years	213 428 (36)
≥55 years	124 097 (21)
Gender, *n* (%)	
Male	299 796 (51)
Female	288 612 (49)
Ethnicity, *n* (%)	
White British	242 614 (41)
Irish	13 745 (2)
Indian	63 999 (11)
Pakistani	35 596 (6)
Bangladeshi	94 643 (16)
Black Caribbean	54 939 (9)
Black African	82 872 (14)
Area‐level deprivation^a^, *n* (%)	
Quintile 5 (most deprived)	370 313 (67)
Quintile 4	147 890 (27)
Quintile 3	28 657 (5)
Quintile 2	5532 (1)
Quintile 1 (least deprived)	2360 (<0.1)
Anti‐psychotic prescriptions, *n* (%)	
Not prescribed anti‐psychotic medication	577167 (98)
Prescribed anti‐psychotic medication	11241 (2)
Severe mental illness, *n* (%)	
No severe mental illness	577638 (98)
Severe mental illness	10770 (2)
Type 2 diabetes mellitus, *n* (%)	
No diabetes mellitus	541541 (92)
Type 2 diabetes mellitus	44622 (8)

a Index of Multiple Deprivation at Lower‐Level Super Output Area.

### Relative risk of Type 2 diabetes

Table 2 shows stratum‐specific estimates for relative risk of Type 2 diabetes in people with severe mental illness, relative to those without severe mental illness, stratified by ethnicity and age, and adjusted for gender and area‐level deprivation (model 1) and gender, area‐level deprivation and anti‐psychotic prescriptions (model 2). Relative risk for the association of severe mental illness with Type 2 diabetes mellitus was strongest for individuals in the 18–34‐year age group, but reduced with increasing age. Adjustment for anti‐psychotic prescriptions only marginally attenuated associations. Trends were similar when age was broken down further into 10‐year bands (Table S1).

**Table 2:** Relative risk (95% CI) of Type 2 diabetes mellitus in people with severe mental illness vs no severe mental illness

	No severe mental illness	Severe mental illness	Age group		
			18–34 years	35–54 years	≥55 years
	With/without Type 2 diabetes, *n*/*n*	With/without Type 2 diabetes, *n*/*n*	Relative risk (95% CI)	Relative risk (95% CI)	Relative risk (95% CI)
Ethnicity					
White British	10 775/22 6175	433/3951	9.81 (5.25, 18.36) *8.77 (4.69, 16.40)*	2.88 (2.42, 3.44) *2.54 (2.13, 3.02)*	1.17 (1.04, 1.31) *1.08 (0.96, 1.21)*
Irish	562/12845	34/249	– *–*	2.84 (1.04, 7.79) *2.50 (0.92, 6.81)*	1.60 (1.16, 2.20) *1.47 (1.07, 2.03)*
Indian	5433/57824	134/482	6.01 (2.32, 15.59) *5.20 (2.01, 13.41)*	2.08 (1.59, 2.72) *1.78 (1.36, 2.34)*	1.13 (0.96, 1.32) *1.02 (0.87, 1.21)*
Pakistani	3071/32073	79/300	5.26 (1.70, 16.26) *4.54 (1.48, 13.99)*	2.14 (1.59, 2.89) *1.81 (1.33, 2.47)*	1.23 (0.99, 1.53) *1.11 (0.88, 1.39)*
Bangladeshi	10 965/82 056	419/1076	7.28 (5.51, 9.63) *6.18 (4.62, 8.28)*	2.02 (1.77, 2.31) *1.71 (1.48, 1.98)*	1.25 (1.14, 1.37) *1.12 (1.03, 1.23)*
Black Caribbean	6427/46 204	406/1596	8.31 (4.16, 16.60) *7.32 (3.66, 14.63)*	2.36(2.01 2.77) *2.06 (1.74, 2.44)*	1.13 (1.02, 1.26) *1.04 (0.94,1.15)*
Black African	5688/75 350	196/1360	3.45 (1.54, 7.76) *3.00 (1.34, 6.73)*	2.13 (1.73, 2.62) *1.85 (1.48, 2.31)*	1.11 (0.90, 1.35) *0.99 (0.81, 1.22)*
Wald test for interaction of ethnicity and severe mental illness, within age group			<0.001	0.02	0.38

–, too few observations to derive estimates.

Model 1: (set in roman) adjusted for gender and area‐level deprivation; Model 2: (set in italic) adjusted for gender, area‐deprivation level, anti‐psychotic prescriptions.

### Prevalence of Type 2 diabetes mellitus in severe mental illness

Overall, the estimated prevalence of Type 2 diabetes mellitus was 16.0% (95% CI 15.1, 16.9) in people with severe mental illness [vs 7.6% (95% CI 7.3, 8.0) in people without severe mental illness] after adjusting for gender and area‐level deprivation. Within each age band, the estimated prevalence of Type 2 diabetes mellitus in people with severe mental illness was 3.3% (95% CI 2.5, 4.0) at age 18–34 years, 14.3% (95% CI 13.0, 15.5) at age 35–54 years and 27.5% (95% CI 25.6, 29.2) at age ≥55 years, after adjusting for gender and area‐level deprivation.

**Figure 2:**
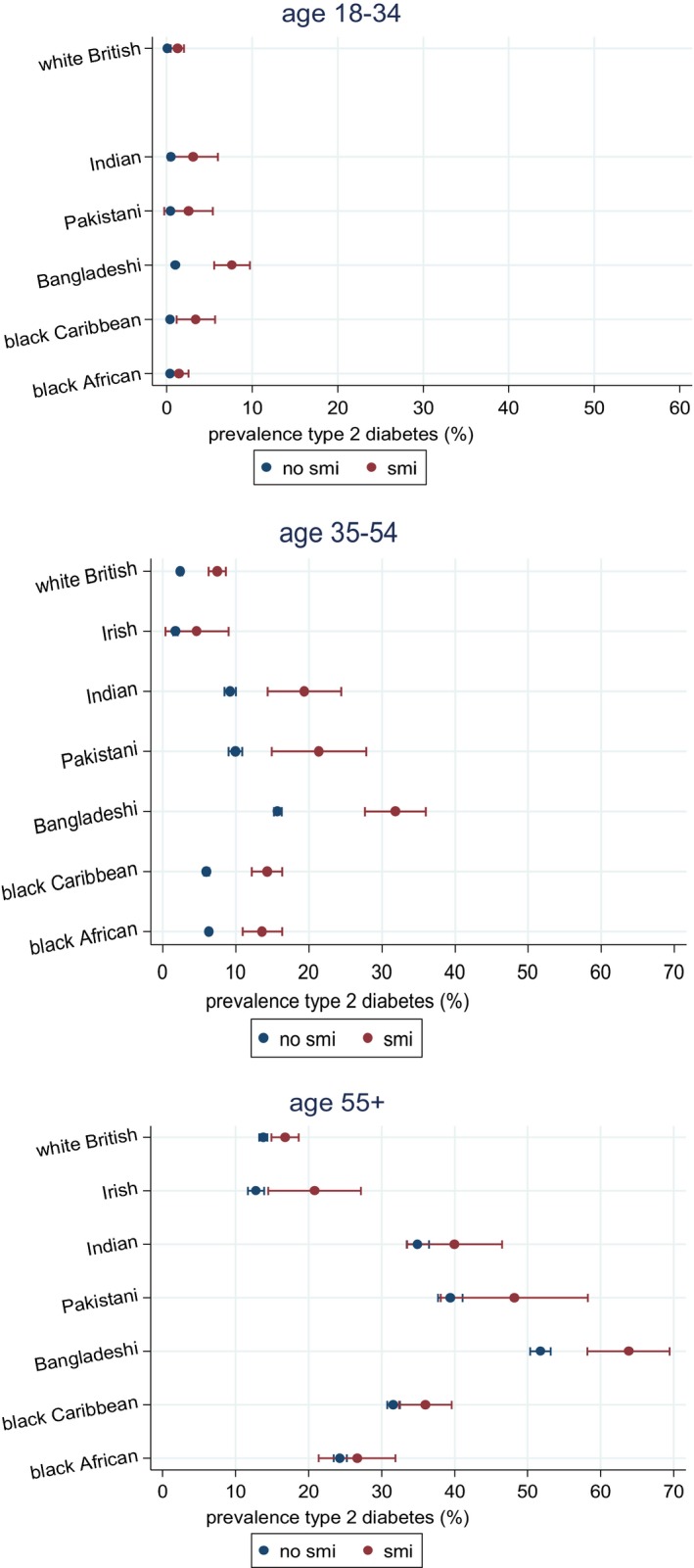
Estimated prevalence of Type 2 diabetes mellitus by severe mental illness, ethnicity and age, adjusted for gender, areal‐deprivation and clustering by practice (Table S3).

In stratified analyses the adjusted estimated prevalence of Type 2 diabetes mellitus was increased in the presence of severe mental illness, across all age and ethnic groups (Fig. 2). Although there was a larger magnitude of risk of Type 2 diabetes mellitus (in relative terms) in the youngest age group (Table 2), absolute estimates of prevalence were most elevated for Bangladeshi people with severe mental illness, who had an estimated prevalence of Type 2 diabetes mellitus of 7.6% (95% CI 5.5–9.6) in the youngest age band (18–34 years); this was 1.0% (95% CI 0.9–1.1) in the Bangladeshi population without severe mental illness (Fig. 2). For the age group 35–54 years, estimated prevalence of Type 2 diabetes mellitus increased further across all ethnic groups living with severe mental illness and was most notable for Indian, Pakistani, Bangladeshi and black Caribbean people with severe mental illness (Table S1 and Fig. 2). For the oldest age group (age ≥55 years) prevalence estimates for Type 2 diabetes mellitus remained elevated in people with severe mental illness across all ethnic groups, but was greatest for Bangladeshi people living with severe mental illness, who had an estimated prevalence of Type 2 diabetes mellitus of 63.8% (95% CI 58.2, 69.4; Table S1). Risk differences are shown in Table S1. In models estimating absolute risk, within the three age bands, there was strong evidence (*P* < 0.001) to indicate that the association of severe mental illness with Type 2 diabetes mellitus varied by ethnicity, with evidence of larger risk differences for each of the ethnic minority groups compared with the white British group (Table S1).

## Discussion

The present study provides confirmatory evidence that the prevalence of Type 2 diabetes mellitus is elevated in people with severe mental illness. The findings also indicated that this was more marked for the ethnic minorities surveyed in this study. Relative to people not known to have severe mental illness, the relative risk of Type 2 diabetes mellitus was most elevated in young populations. In models estimating absolute risk, estimated prevalence of Type 2 diabetes mellitus in people with severe mental illness was elevated in most ethnic minority groups and especially marked in Bangladeshi, Indian and Pakistani people. Although adjustment for anti‐psychotic prescriptions attenuated some of the association, on the whole, most of the associations persisted.

The findings are in keeping with previous work which has shown a strong association between severe mental illness and Type 2 diabetes mellitus, not fully accounted for through anti‐psychotic prescribing 1, 5, 6. Previous studies have suggested the risk of Type 2 diabetes mellitus in people with severe mental illness may be 2–4 times higher than in the background population 30. Although this magnitude of association was confirmed in the present study for people aged 35–54 years, in the youngest age group of 18–34 years, the relative risk of Type 2 diabetes mellitus ranged between 3‐ and 10‐fold, by age 55 years the relative risk for association of severe mental illness with Type 2 diabetes mellitus was diminished across all ethnic groups. Life expectancy of people with severe mental illness is much reduced 1, therefore, the findings may indicate a healthy survivor effect among those with severe mental illness. A similar trend has been demonstrated previously for cardiovascular and stroke mortality in people with severe mental illness 30. Findings may also reflect competing risks 31, in other words, the increased risk of premature death from related causes removes people from the ‘at‐risk’ (severe mental illness) population, leading to a reduced relative risk of Type 2 diabetes in people with severe mental illness in the oldest age groups. Future work using longitudinal data linked to mortality records could be used to understand this further. Another factor that may have accounted for these findings is the fact that Type 2 diabetes mellitus is relatively rare in young healthy populations (hence we observed higher relative risks in the population with severe mental illness relative to the populations without severe mental illness in the younger age group)^4^; the steep rise in Type 2 diabetes mellitus prevalence at older ages among those without severe mental illnesses may have made larger relative risks in the severe mental illness population less likely.

Findings from the additive models of risk, illustrating differences in the prevalence of Type 2 diabetes mellitus, indicated that ethnic minority groups are more likely to have Type 2 diabetes mellitus in the presence of severe mental illness compared with white British people with severe mental illnesses. These differences were marked; for example, by age ≥55 years, whereas white British individuals with severe mental illness had a prevalence of Type 2 diabetes mellitus of 16.8% [95% CI 14.9, 18.6 (compared with white British people without severe mental illnesses among whom the prevalence was 13.8% (95% CI 13.2, 14.4)], in the Bangladeshi group with severe mental illness this was 63.8% [95% CI 58.2, 69.4 (compared with 51.7% (95% CI 50.3, 53.1) in the Bangladeshi group without severe mental illness (Fig. 2)]. Assessment of effect modification on an additive scale suggested that the combined effect of ethnicity and severe mental illness on Type 2 diabetes mellitus risk within age bands was greater than the sum of the individual effects (Table S2).

The relative risk for the association of severe mental illness with Type 2 diabetes mellitus was elevated and persisted across all ethnic groups, despite adjustment for anti‐psychotic medications. The finding of an increased risk of Type 2 diabetes mellitus in severe mental illness, which persisted despite adjustment for anti‐psychotic prescriptions, suggests other factors may operate which increase the risk of diabetes in severe mental illnesses. This may include the impact of severe mental illnesses on physical health and ability to access preventative healthcare, relevant to all people with severe mental illnesses, irrespective of ethnicity.

The present analyses are based on a large primary care database, covering 98% of practices in a well‐defined ethnically diverse location in the UK. This population is likely to be representative of other ethnically diverse regions in inner cities and could be generalized to other similar contexts. Local initiatives to improve the recording of self‐ascribed ethnicity meant that this variable was relatively complete. The large sample size with relatively complete encoding for self‐ascribed ethnicity meant that it was possible to assess differences in prevalence estimates of Type 2 diabetes mellitus without recourse to grouped categories (e.g. ‘South Asian’ or ‘Black’). Using such an approach highlighted intra‐ethnic differences in the prevalence of Type 2 diabetes mellitus with the comorbidity of severe mental illness. A limitation is that we did not have information relating to country of birth and family origins, which may have permitted a more nuanced assessment of ethnicity 32.

Previous studies have highlighted the fact that a high proportion of people with severe mental illness may have undetected diabetes mellitus 4. At the time of this study, screening for Type 2 diabetes mellitus in people aged > 40 years with severe mental illness was still financially incentivised nationally, with high rates of completion (e.g. 82.8% completion in the London area; https://www.gpcontract.co.uk/browse/08K/Mental%20Health/13). Given this high response rate, a relative strength of the present study is that the prevalence estimates of Type 2 diabetes mellitus in the population with severe mental illness are likely to have been relatively accurate in those aged >40 years; however, it is possible that rates of diagnosis may have been lower in individuals with severe mental illnesses aged <40 years, because this was not incentivised. Despite this, the detected prevalence of Type 2 diabetes mellitus remained appreciably higher in the youngest age group, which is a concern as this may have been an underestimate. Although a healthy survivor effect could account for the findings in the oldest age group, the cross‐sectional nature of this dataset means that it is not possible to be certain about this, nor the temporal association of severe mental illness and diabetes. The differential association of severe mental illness with Type 2 diabetes mellitus by age could have been accentuated by ascertainment biases, as incident Type 2 diabetes mellitus may have been less likely to have been ascertained in older people with severe mental illness as there may be less attention to medication side effects in this age group, especially if people had been on a stable regime for long periods of time. It is also possible that older people with chronic mental disorders are less likely to visit GPs, complain of relevant symptoms, or have family members who can assist and advocate for them, which could have also led to a lower reported prevalence of Type 2 diabetes mellitus in this group. The prevalence estimates may have been residually confounded by social deprivation over and above the area‐level deprivation measures.

Although we adjusted for anti‐psychotic medication prescriptions, most associations persisted. We could not adjust for BMI because of high levels of missing data for this variable. Future research should consider this and other mediators, potentially on the causal pathway, preferably using longitudinal data.

Efforts to concentrate case‐finding and management should include Type 2 diabetes mellitus screening in younger populations with severe mental illness. A previous systematic review indicated that the prevalence of Type 2 diabetes mellitus is elevated in ethnic minority groups across European settings (especially in South Asian, Middle Eastern and North African, Sub‐Saharan African and South/Central American populations 12). The findings of the present study support a similar trend, but importantly, indicate that some ethnic minority groups may be even more likely to have Type 2 diabetes mellitus with the additional presence of severe mental illness.

In conclusion, these findings potentially inform current discussions on screening for diabetes mellitus in severe mental illness, particularly in younger populations and in areas which are ethnically diverse. Screening should not just be restricted to people prescribed anti‐psychotic medications. Current debates around screening for Type 2 diabetes mellitus in severe mental illness will also need to be informed by evidence of benefit from screening. The findings also have implications for the clinical care of all individuals living with severe mental illnesses as, irrespective of ethnicity, Type 2 diabetes mellitus is more prevalent.

## Funding sources

J.M. is a Clinician Scientist funded by the Health Foundation, working with the Academy of Medical Sciences. C.M. is supported by a European Research Council Consolidator Award (Ref: ERC‐CoG‐2014 ‐ Proposal 648837, REACH). P.S. is a population health scientist funded by the Medical Research Council (MR/K021494/1). G.T. is supported by the National Institute for Health Research (NIHR) Collaboration for Leadership in Applied Health Research and Care South London at King's College London Foundation Trust. G.T. acknowledges financial support from the Department of Health via the National Institute for Health Research (NIHR) Biomedical Research Centre and Dementia Unit awarded to South London and Maudsley NHS Foundation Trust in partnership with King's College London and King's College Hospital NHS Foundation Trust. G.T. is supported by the European Union Seventh Framework Programme (FP7/2007‐2013) Emerald project. M.A. was funded/supported by the National Institute for Health Research (NIHR) Biomedical Research Centre based at Guy's and St Thomas' NHS Foundation Trust and King's College London. The views expressed are those of the author(s) and not necessarily those of the NHS, the NIHR, the Department of Health, or funders.

## Competing interests

All authors except F.G. and I.P. declare no financial relationships with any organizations that might have an interest in the submitted work in the previous three years; no other relationships or activities that could appear to have influenced the submitted work. F.G. has received honoraria for advisory work and lectures from Roche, Lundbeck, Otsuka and Sunovion and has a family member with professional links to Lilly and GSK, who has shares in GSK. F.G. has also joined a research team supported by an NHS innovation grant, supported by Janssen. I.P. supervises a PhD student funded by Novo Nordisk.

## Supporting information


**Table S1.** Relative risk (95% CI) for Type 2 diabetes mellitus in people with severe mental illness vs. no severe mental illness; stratified by ethnicity and age (ten year bands).
**Table S2.** Risk difference (RD) with 95% Confidence Intervals for estimated prevalence of Type 2 diabetes mellitus in people with severe mental illness compared to people without severe mental illness.

**Table S3.** Estimated prevalence of Type 2 diabetes mellitus across ethnic groups, by age and severe mental illness status.
