# Supplementary material for: Type 2 diabetes mellitus in people with severe mental illness: inequalities by ethnicity and age. Cross‐sectional analysis of 588 408 records from the UK
Source: Diabet Med. 2017 Jan 30;34(7):916–24. doi: 10.1111/dme.13298 (PMC5484374; doi:10.1111/dme.13298)
Supplement: Supplementary file 1 — Table S1. Relative risk (95% CI) for Type 2 diabetes mellitus in people with severe mental illness vs. no severe mental illness; stratified by ethnicity and age (ten year bands). Table S2. Risk difference (RD) with 95% Confidence Intervals for estimated prevalence of Type 2 diabetes mellitus in people with severe mental illness compared to people without severe mental illness. [file DME-34-916-s001.docx]

**APPENDIX/ ONLINE SUPPLEMENT**

**TABLE S1:** Relative risk (95% CI) for Type 2 diabetes mellitus in people with severe mental illness vs. no severe mental illness; stratified by ethnicity and age (ten year bands)

|  |  | **Age group** |  |  |  |  |  |  |
| --- | --- | --- | --- | --- | --- | --- | --- | --- |
|  |  | **18-24** | **25-34** | **35-44** | **45-54** | **55-64** | **65-74** | **75+** |
|  |  |  |  |  |  |  |  |  |
| **Ethnicity** | **Row**  **totals** | **Relative risk (95% CI)** | **Relative risk (95% CI)** | **Relative risk (95% CI)** | **Relative risk (95% CI)** | **Relative risk (95% CI)** | **Relative risk (95% CI)** | **Relative risk (95% CI)** |
| White British | 241334 | 19.55 (2.31, 165.21) | 8.83 (4.64, 16.81) | 4.42 (3.04, 6.44) | 2.21 (1.80, 2.71) | 1.46 (1.21, 1.76) | 1.13 (0.94, 1.39) | 0.97 (0.77, 1.23) |
| Irish | 13690 | - | - | 7.10 (0.97, 51.86) | 1.78 (0.53, 5.95) | 2.09 (1.17, 3.74) | 1.60 (0.89, 2.87) | 1.31 (0.71, 2.41) |
| Indian | 63873 | - | 5.54 (2.12, 14.50) | 2.86 (1.62, 5.05) | 1.51 (1.16, 1.98) | 1.33 (1.07, 1.65) | 0.95 (0.66, 1.37) | 0.95 (0.62, 1.45) |
| Pakistani | 35523 | - | 4.98 (1.60, 15.54) | 3.14 (1.90, 5.18) | 1.45 (1.01, 2.07) | 1.25 (0.90, 1.74) | 1.39 (0.96, 2.00) | 1.01 (0.57, 1.78) |
| Bangladeshi | 94516 | - | 6.39 (4.84, 8.45) | 2.43 (2.01, 2.92) | 1.57 (1.38, 1.78) | 1.23 (1.07, 1.41) | 1.35 (1.19, 1.54) | 0.97 (0.69, 1.37) |
| Black Caribbean | 54633 | - | 7.37 (3.66, 14.83) | 3.28 (2.31, 4.66) | 2.06 (1.70, 2.50) | 1.47 (1.21, 1.78) | 1.07 (0.91, 1.25) | 1.06 (0.91, 1.23) |
| Black African | 82594 | 9.55 (1.46, 62.35) | 2.75 (1.15, 6.56) | 2.37 (1.68, 3.33) | 1.94 (1.56, 2.40) | 0.99 (0.73, 1.34) | 1.44 (1.08, 1.91) | 0.56 (0.22, 1.40) |
| P value (Wald tests)* |  | - | *P*<0.001 | *P*=0.001 | *P*=0.06 | *P*=0.18 | p=0.42 | p=0.94 |

***Key:*** *Adjusted for gender and area-level deprivation; - too few observations to derive estimate;*Wald tests to assess strength of evidence for ethnicity as an effect modifier in the association between severe mental illness and Type 2 diabetes mellitus, 2-way interactions (ethnicity*severe mental illness) within each age-band*

**TABLE S2:** Risk difference (RD) with 95% Confidence Intervals for estimated prevalence of Type 2 diabetes mellitus in people with severe mental illness compared to people without severe mental illness

|  | **No Severe mental illness** | **Severe mental illness** | **Age 18-34** | **Age 35-54** | **Age 55+** |
| --- | --- | --- | --- | --- | --- |
|  | **N with/without Type 2 diabetes** | **N with/without Type 2 diabetes** | **RD (95% CI)** | **RD (95% CI)** | **RD (95% CI)** |
| **Ethnicity** |  |  |  |  |  |
| White British | 10775/226175 | 433/3951 | 1.13 (0.36, 1.91) | 4.71 (3.51, 5.91) | 2.57 (0.75, 4.39) |
| Irish | 562/12845 | 34/249 | - | 2.61 (-1.71, 6.92) | 8.08 (1.69, 14.47) |
| Indian | 5433/57824 | 134/482 | 2.59 (-0.31, 5.50) | 10.06 (5.00, 15.12) | 4.97 (-1.34, 11.28) |
| Pakistani | 3071/32073 | 79/300 | 2.08 (-0.75, 4.92) | 11.40 (5.08, 17.73) | 8.94(-1.49, 19.38) |
| Bangladeshi | 10965/82056 | 419/1076 | 6.62 (4.52, 8.72) | 16.08 (11.94, 20.22) | 12.38 (6.92, 17.84) |
| Black Caribbean | 6427/46204 | 406/1596 | 3.02 (0.78, 5.26) | 8.18 (6.06, 10.30) | 4.30 (0.63, 7.97) |
| Black African | 5688/75350 | 196/1360 | 1.04 (-0.08, 2.16) | 7.26 (4.54, 9.99) | 2.64 (-2.67, 7.95) |
| *Wald test for interaction of ethnicity and severe mental illness within age group* |  |  | *p<0.001* | *p<0.001* | *p=0.02* |

*Risk differences have been adjusted for gender, area-level deprivation and practice-level clustering.*
