# Supplementary material for: Type 2 diabetes mellitus in people with severe mental illness: inequalities by ethnicity and age. Cross‐sectional analysis of 588 408 records from the UK
Source: Diabet Med. 2017 Jan 30;34(7):916–24. doi: 10.1111/dme.13298 (PMC5484374; doi:10.1111/dme.13298)
Supplement: Supplementary file 2 — Table S3. Estimated prevalence of Type 2 diabetes mellitus across ethnic groups, by age and severe mental illness status. [file DME-34-916-s002.docx]

**Supplementary table (to accompany Figure 2):**

Estimated prevalence of type 2 diabetes mellitus across ethnic groups, by age and severe mental illness status*

| **Age group** | **Ethnicity/ SMI status** | **Prevalence**  **%** | **95%**  **Confidence Interval** |
| --- | --- | --- | --- |
| **18-34 years** | White British no SMI | 0.1 | (0.1, 0.2) |
|  | **White British SMI** | **1.3** | **(0.5, 2.0)** |
|  | Indian no SMI | 0.5 | (0.4, 0.6) |
|  | **Indian SMI** | **3.1** | **(0.2, 6.0)** |
|  | Pakistani no SMI | 0.5 | (0.4, 0.6) |
|  | **Pakistani SMI** | **2.6** | **(-0.3, 5.4)** |
|  | Bangladeshi no SMI | 1.0 | (0.9, 1.1) |
|  | **Bangladeshi SMI** | **7.7** | **(5.5, 9.8)** |
|  | Black Caribbean no SMI | 0.4 | (0.3, 0.5) |
|  | **Black Caribbean SMI** | **3.4** | **(1.2, 5.7)** |
|  | Black African no SMI | 0.4 | (0.3, 0.5) |
|  | **Black African SMI** | **1.5** | **(0.3, 2.6)** |
|  |  |  |  |
| **35-54 years** | White British no SMI | 2.4 | (2.2, 2.6) |
|  | **White British SMI** | **7.4** | **(6.2, 8.7)** |
|  | Irish no SMI | 1.8 | (1.4, 2.1) |
|  | **Irish SMI** | 4.7 | (0.4, 9.0) |
|  | Indian no SMI | 9.2 | (8.5, 10.0) |
|  | **Indian SMI** | **19.4** | **(14.4, 24.4)** |
|  | Pakistani no SMI | 9.9 | (9.0, 10.9) |
|  | **Pakistani SMI** | **21.4** | **(14.9, 27.8)** |
|  | Bangladeshi no SMI | 15.7 | (15.2, 16.3) |
|  | **Bangladeshi SMI** | **31.8** | **(27.7, 36.0)** |
|  | Black Caribbean no SMI | 6.0 | (5.6, 6.4) |
|  | **Black Caribbean SMI** | **14.3** | **(12.2, 16.4)** |
|  | Black African no SMI | 6.3 | (6.1, 6.6) |
|  | **Black African SMI** | **13.6** | **(10.9, 16.3)** |
|  |  |  |  |
| **55+ years** | White British no SMI | 13.8 | (13.2, 14.4) |
|  | **White British SMI** | **16.8** | **(14.9, 18.6)** |
|  | Irish no SMI | 12.8 | (11.7, 13.9) |
|  | **Irish SMI** | **20.8** | **(14.5, 27.1)** |
|  | Indian no SMI | 34.9 | (33.4, 36.5) |
|  | **Indian SMI** | **40.0** | **(33.5, 46.5)** |
|  | Pakistani no SMI | 39.4 | (37.7, 41.1) |
|  | **Pakistani SMI** | **48.2** | **(38.1, 58.2)** |
|  | Bangladeshi no SMI | 51.7 | (50.3, 53.1) |
|  | **Bangladeshi SMI** | **63.8** | **(58.2, 69.4)** |
|  | Black Caribbean no SMI | 31.6 | (30.7, 32.5) |
|  | **Black Caribbean SMI** | **36.0** | **(32.4, 39.6)** |
|  | Black African no SMI | 24.3 | (23.4, 25.2) |
|  | **Black African SMI** | **26.6** | **(21.4, 31.9)** |

***Key:*** **All estimates have been adjusted for gender, area-level deprivation and take into account clustering by GP practice. ‘SMI’ Severe Mental Illness*
